# Femoral prosthesis neck fracture following total hip arthroplasty — a systematic review

**DOI:** 10.1186/s42836-020-00047-3

**Published:** 2020-10-16

**Authors:** P. G. van Doesburg, E. J. van Langelaan, I. Apachitei, M. R. Bénard, S. H. M. Verdegaal

**Affiliations:** 1 Department of Orthopaedic Surgery, Alrijne Hospital Leiderdorp, Simon Smitweg 1, 2353GA Leiderdorp, The Netherlands; 2 Biomechanical Engineering Department Biomaterials & Tissue Biomechanics Section, Delft University of Technology, Delft, The Netherlands

**Keywords:** Total hip arthroplasty, Fatigue fracture, Trunnionosis, Corrosion

## Abstract

**Purpose:**

Head-neck modularity was introduced into total hip arthroplasty to provide more intraoperative surgical options. However, modularity led to new problems, such as trunnionosis and fractures of the femoral prosthesis neck.

The purpose of this study was to identify risk factors for hip neck fractures and to provide recommendations to prevent damage and fractures of the neck.

**Methods:**

A systematic review of the literature was performed according to the PRISMA guidelines.

**Results:**

Thirty-three case studies were included. Methodologically, most included studies were of moderate or good quality. The 80 neck fractures included in the review took place, on average, 7 years after stem placement. Male gender, high body weight, obesity, previous revision surgery, mixing components from different manufacturers, use of long skirted heads, cobalt-chromium (large size) heads were identified as potential risk factors.

**Conclusion:**

Hip neck fractures occur on average 7 years after stem placement. The etiology of hip neck fractures is multifactorial. This review revealed several preventable implant- and surgeon-related risk factors.

## Introduction

Hip stem and hip neck fractures were well-known complications of total hip arthroplasty (THA) in the past [1–4]. Better quality stainless steel and the use of cobalt-chromium stems reduced this problem. However, after the introduction of modularity, neck fractures are again a growing field of interest.

In contrast to the beneficiary aspects, modularity has also led to new problems at the head-neck junction. Specific risk factors are wear at the head-neck connection, adverse local tissue reactions and gross trunnion failures (trunnion deformation, head disassociation and fatigue fracture). Neither the problem of corrosion was recognized, nor was it seen as a major problem. Recent studies showed that up to 3% of all total hip revisions were performed because of corrosion at the head-neck connection (trunnionosis) [5]. Fatigue fractures of hip necks are rare but recently mounting cases were published.

**Fig. 1:**
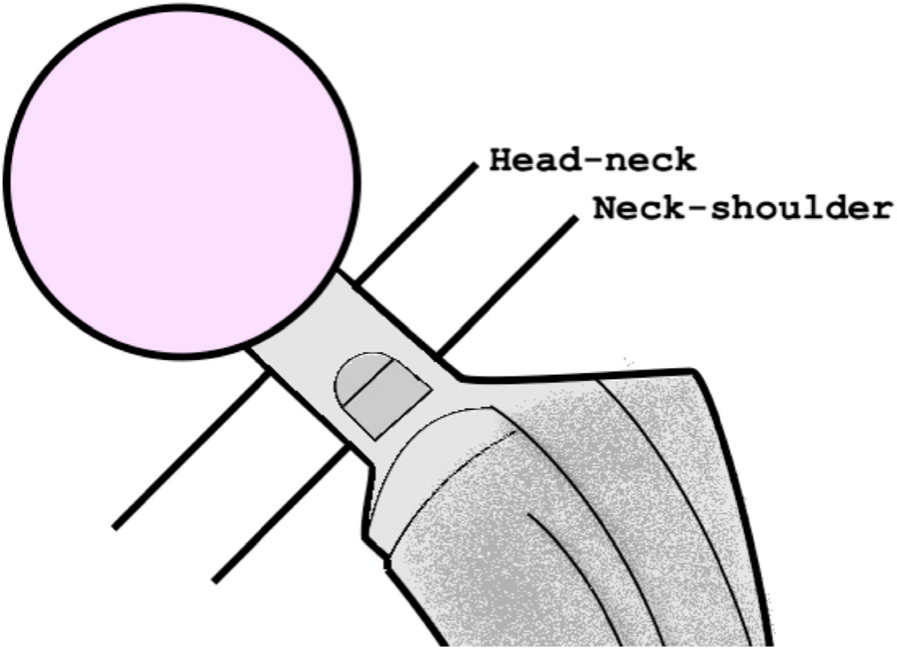
Two types of hip neck fractures: head-neck fractures are located at the proximal part of the neck and neck-shoulder fractures at the distal part of the neck

Hip neck fatigue fractures can be divided into head-neck fractures and neck-shoulder fractures (Fig. 1). Head-neck fractures are located at the proximal part of the neck while neck-shoulder fractures at the distal part of the neck. A head-neck fracture is an example of gross trunnion failure and is possibly caused by a cascade starting with fretting at the head-neck coupling leading to damage of the metal by corrosion [6]. The loss of material could lead to a fatigue fracture of the neck. In contrast, a neck-shoulder fracture is not related to the modular connection.

Literature on the exact etiology and prevention of neck fractures is scarce. In the coming years, more patients will receive THA at a relatively young age, leading to more revision surgery and stems staying in place for a longer period. Therefore, more problems at the trunnion, including fractures, are expected in the not-very-distant future.

The purpose of our study was to identify potential risk factors for the development of neck fractures, and to provide future treatment recommendations in primary hip arthroplasty and hip revision surgery to prevent damage of the neck.

## Materials and methods

### Literature search

A systematic review of the literature was performed according to the PRISMA guidelines [7]. All studies on neck fractures in total hip arthroplasty were searched in PubMed, Web of Science, and EMBASE. All databases were searched using the following terms: hip arthroplasty, taper, trunnion, cone, fracture, prosthetic failure, neck fracture hip stem. The references in each included study were searched for additional eligible studies.

### Study selection

All published studies on hip stem neck fractures in English, German, French and Dutch were included. Monoblock hip prosthesis, modular neck hip prosthesis, animal and cadaver studies were excluded. Two review authors independently screened all titles or abstracts. Any obviously irrelevant studies were ruled out. Full-text reports were obtained for the remaining potentially relevant studies. From the remaining studies, all included neck fractures were divided into two groups based on the localization of the fracture, neck-shoulder region fractures and head-neck region. Any disagreement about the type of fracture and inclusion that arose between the reviewers was resolved by discussion.

### Quality assessment

Methodological quality was evaluated using a critical appraisal instrument as described by Murad et al adapted for case reports/series (Table 1) [8]. Two reviewing authors independently performed this critical appraisal of the included articles and scored the overall methodological quality (good, moderate or bad). Any disagreement about the quality that arose between the reviewers was discussed. The methodological quality was assessed in terms of selection, ascertainment, causality and reporting.

**Table 1:** Critical appraisal instrument

Study		
Domains	Questions	Answer
Selection	1. Does the patient(s) represent(s) the whole experience of the investigator (center) or is the selection method unclear to the extent that other patients with similar presentation may not have been reported?	
Ascertainment	2. Was the outcome adequately ascertained?	
Causality	3. Were other alternative causes that may explain the observation ruled out?	
	4. Was follow-up long enough for outcomes to occur?	
	5. Was there further analysis done to investigate the cause of fracture?	
Reporting	6. Is the case(s) described with sufficient details to allow other investigators to replicate the research or to allow practitioners make inferences related to their own practice?	
**Overall score**	– or +/− or +	

## Results

### Search results

**Fig. 2:**
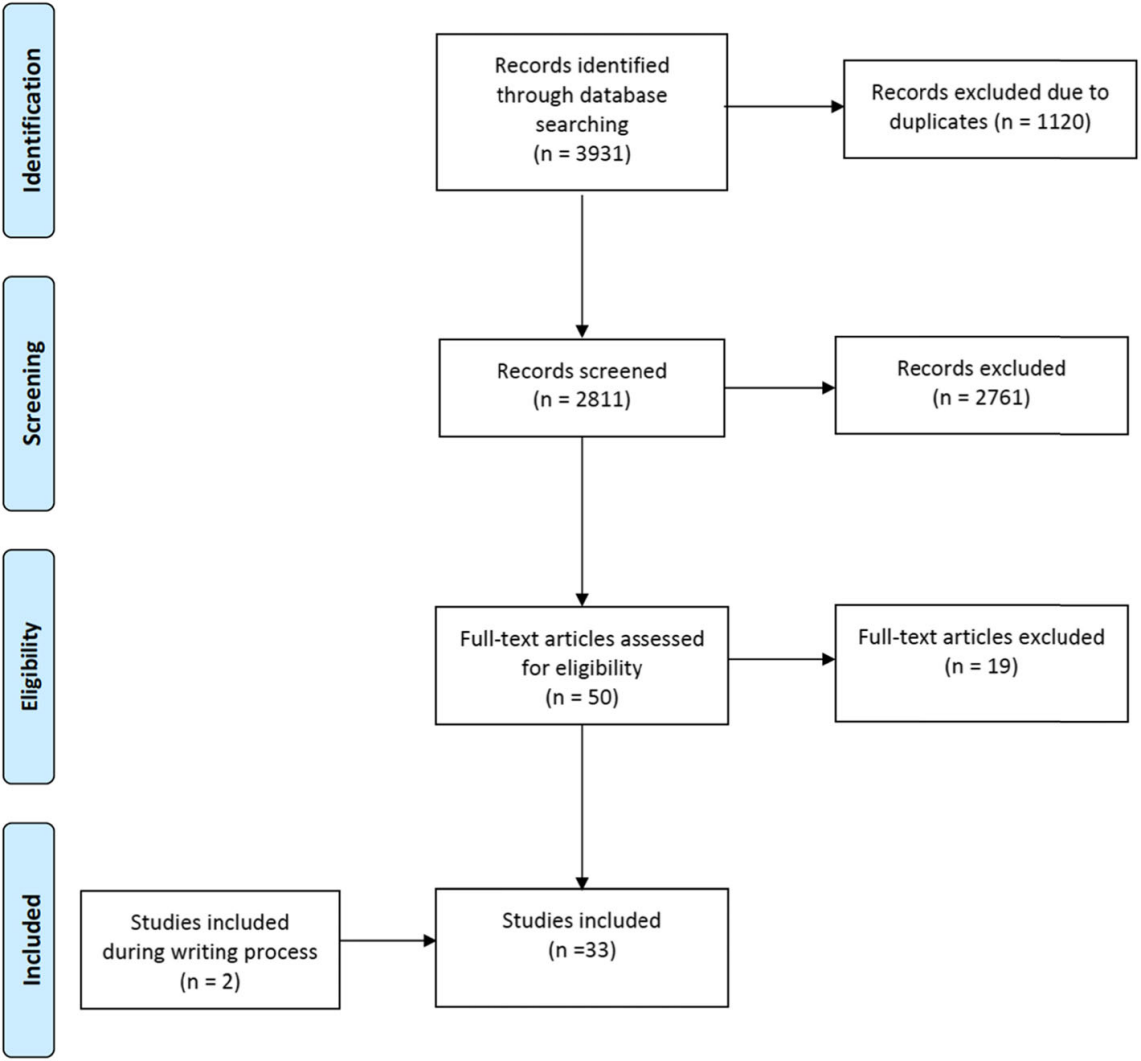
PRISMA flowchart of the review process of our study

The literature search yielded 3933 potentially-relevant studies (Fig. 2). After removal of duplicates, 2813 studies were left for review. After review of the titles and abstracts with exclusion of non-relevant studies, a total of 52 full-text articles were assessed further for eligibility. After a full-text review, 33 studies were included for the final analysis (Fig. 2). All studies were case reports or case series of moderate or good methodological quality in most studies (*n* = 28) (Table 2).

**Table 2:** Characteristics of the 33 studies included

	Study	Year	Number of fractures	Fracture location	Mean age (y) - weight (kg) - BMI (kg/m^2^)	Gender (M – F)	Type of stem	Methodological score
1	Peterson	2019	1	Head-neck: 1	45 y - 94 kg - 27 kg/m^2^	1–0	C-taper stem	+
2	Takai	2019	1	Neck-shoulder: 1	57 y – 70 kg – 27 kg/m^2^	0–1	AHFIX Q	+
3	Ryniewicz	2018	1	Neck-shoulder: 1	73 y -? -?	1–0	Aura II	+
4	Bolland	2016	26	Head-neck: 20 Neck-shoulder: 6	69 y - 107 kg -?	20–6	Exeter	+/−
5	Facek	2016	1	Neck-shoulder: 1	70 y - 71 kg -?	1–0	Exeter	+
6	Morlock	2016	3	Head-neck: 3	63 y - 109 kg - 33 kg/m^2^	3–0	BiMetric	+
7	Reito	2015	3	Head-neck: 3	79 y - 99 kg - 32 kg/m^2^	2–1	Exeter V40	+/−
7	Spanyer	2015	2	Head-neck:1 Neck-shoulder: 1	52 y -? - 28 kg/m^2^	2–0	Accolade1	+/−
8	Yoshimoto	2015	2	Neck-shoulder: 2	58 y - 74 kg - 25 kg/m^2^	2–0	Kyocera	+
10	Banerjee	2014	1	Head-neck: 1	55 y - 81 kg - 47 kg/m^2^	1–0	RMHS	+/−
11	Baratz	2014	1	Head-neck: 1	73 y - 89 kg - 32 kg/m^2^	1–0	Meridian	+/−
12	Hamlin	2014	1	Head-neck: 1	76 y – 141 kg - 45 kg/m^2^	1–0	Exeter	+/−
13	Jang	2013	1	Neck-shoulder: 1	74 y - 107 kg - 35 kg/m^2^	1–0	Corail	+
14	Lizano-Diez	2013	1	Head-neck: 1	67 y - 110 kg - 32 kg/m^2^	1–0	Bicontact	+/−
15	Nganbe	2013	1	Neck-shoulder: 1	75 y -? -?	1–0	Lord	+
16	Haasper	2012	2	Neck-shoulder: 2	50 y -? -?	1–1	Collum Femoris Preserving (CFP)	+/−
17	Morley	2012	1	Head-neck: 1	53 y - 110 kg - 32 kg/m^2^	1–0	C-stem	+/−
18	Bos	2011	1	Head-neck: 1	58 y - 92 kg - 30 kg/m^2^	1–0	Elite Plus	+/−
19	Garg	2011	1	Head-neck: 1	31 y - 78 kg - 26 kg/m^2^	1–0	Anatomic Medullary Locking	–
20	O’neill	2011	1	Neck-shoulder: 1	76 y -? - 28 kg/m^2^	0–1	Exeter revision	–
21	Unnanuntana	2010	2	Head-neck: 2	60 y - 88 kg - 29 kg/m^2^	2–0	Anatomic Medullary Locking	+
22	Lam	2008	4	Head-neck: 4	80 y - 103 kg - 34 kg/m^2^	? -?	Omnifit	+/−
23	Briant-Evans	2007	2	Neck-shoulder: 2	55 y -? - 39 kg/m^2^	0–2	Corin Eurocone Taper-Fit CDH	+/−
24	Grivas	2007	1	Neck-shoulder: 1	68 y - 70 kg - 26 kg/m^2^	1–0	SEM3	+
25	Harvie	2007	1	Neck-shoulder: 1	73 y - 90 kg - 30 kg/m^2^	1–0	JRI-Furlong	+
26	Botti	2005	1	Head-neck: 1	85 y - 90 kg - 28 kg/m^2^	1–0	Anatomic Medullary Locking	+/−
27	Morgan-Hough	2004	1	Neck-shoulder: 1	64 y - 74 - 24 kg/m^2^	1–0	JRI Limited	+
28	Vatani	2002	9	Head-neck: 9	62 y - 79 kg - 26 kg/m^2^	8–1	Modular Charnley by Medical Tec.	+
29	Lee	2001	2	Neck-shoulder: 2	65 y - 104 kg - 33 kg/m^2^	2–0	Exactech Opteon	+/−
30	Allcock	1997	1	Neck-shoulder: 1	52 y - 90 kg - 26 kg/m^2^	1–0	BHC prosthesis	–
31	Artime	1997	1	Head-neck: 1	37 y -? -?	1–0	Lord	–
32	Gilbert	1994	2	Head-neck: 2	65 y - 112 kg - 36 kg/m^2^	2–0	PCA	+
33	Barrack	1993	1	Head-neck:1	66 y - 91 kg - 27 kg/m^2^	1–0	Osteonics	–
			80	Head-neck: 55 Neck-shoulder: 25	65 y - 98 kg - 30 kg/m^2^	63–13 (4?)		

### Study characteristics

The characteristics of the included studies are presented in Table 2. Overall, 80 fractures were reported, with the patients having a mean age of 65 years (SD 10.0 years), a mean weight of 94 kg (SD 15.1 kg) and a mean BMI of 31 kg/m^2^ (SD 5.7 kg/m^2^). Fifty-five of the fractures were head-neck fractures and 25 neck-shoulder fractures.

### Head-neck fractures

Fifty-five head-neck region fractures were identified in 55 patients with a mean age of 66 years (SD 10.5 years), mean weight of 99 kg (SD 15.3 kg) and a mean BMI of 32 kg/m^2^ (SD 6.3 kg/m^2^). Twenty-nine patients were male (in 24 fractures, the gender was not reported). Fifty-two patients had a spontaneous fracture. Only three patients had a trauma just before the fracture. Two patients fell from standing height. The third patient was struck by lightning while walking. The mean time of fracture after THA was 7 years (SD 4.3 years). Eighteen patients had signs of corrosion and seven stems showed inter-granular corrosion at the time of fracture. Skirted heads or large-size femoral heads (> 40 mm) were used in 20 patients. Five patients had revision surgery prior to the fracture, leaving the original stem in place. However, not all studies provided detailed information about previous revision surgeries. Stems were made from stainless steel (*n* = 34), titanium (*n* = 8) or cobalt-chrome (*n* = 8) and most femoral heads were cobalt-chrome (*n* = 21). In 31 cases the material of the femoral head was not reported.

### Neck-shoulder fractures

Twenty-five neck-shoulder region fractures were identified in 25 patients with a mean age of 64 years (SD 8.8 years), mean weight of 94 kg (SD 16.2 kg) and a mean BMI of 29 kg/m^2^ (SD 4.7 kg/m^2^). Fourteen patients were male (in six patients, the gender was not reported). All patients had a sudden onset of hip pain, without a prior traumatic event. The neck fractured on average 6 years (SD 4.2 years) after implantation of the femoral stem. Three patients had a previous revision, with at least head exchange. Nine stems fractured through the introducer hole, four fractures were caused by laser etching, and 3 fractures were caused by excessive stress on a (sharp) corner of the prosthesis. Stems were made from stainless steel (*n* = 11), titanium (*n* = 10) or cobalt-chrome (*n* = 3). Femoral heads were made of cobalt-chrome (*n* = 11) and ceramics (zirconic *n* = 4, others *n* = 2). In 8 cases, the material of the femoral head was not reported.

## Discussion

Neck fracture following total hip arthroplasty is a rare complication. Neck fractures are expected to occur more frequently, as more and more young patients will undergo THA. Most of these patients will have their hip stem in place for a long period. Young patients receiving THA will probably have to receive revision at some time point. The systematic review provides an overview on the available literature, in both primary and revision cases. Neck fractures occurred on average 7 years (SD 4.2 years) after hip stem placement. Several risk factors, both in head-neck fractures and neck-shoulder fractures, were identified.

Neck fracture is etiologically multifactorial and this study identified some potential risk factors. In the head-neck region group, (mechanically-assisted crevice) corrosion and the use of cobalt-chromium long-skirted or large-size femoral heads were frequently reported. Trunnionosis in the head-neck group might play an important role. Neck-shoulder fracture was frequently associated with some specific implant-related characteristics, such as introducer holes, sharp etches and laser etching. Several implant-, patient-, and surgeon-related factors could increase the risk of neck fractures. Two fractures were located in mid-neck and these fractures were added into the neck-shoulder group [9, 10]. It must be mentioned that this classification is arbitrary.

### Implant-related factors

The most commonly reported implant-related risk factors were the use of large-size heads (> 40 mm), skirted heads, corrosion and design flaws. Crevice corrosion is caused by a cascade starting with fretting at the head-neck coupling leading to wear and disruption of the passive oxide layer. Severe corrosion caused by fretting results in reduced contact between the head-neck connection and leads to channels for fluid ingress followed by a stagnant body fluid in the crevice. Here, a chemical reaction takes place, forming hydrogen chloride. The hydrogen chloride decreases the local solution pH, damaging the metal, causing loss of material and pits at the trunnion [6]. The material loss could possibly lead to a fatigue fracture of the neck. Previous studies have identified several risk factors for corrosion. An important risk factor for corrosion was the use of cobalt-chromium alloy femoral heads on a titanium or stainless steel trunnion. Growing evidence in literature showed that ceramic heads reduced this risk [9]. Nonetheless, cobalt-chromium alloy femoral heads are still most frequently employed in total hip arthroplasty. In the last decade in Europe, there has been a trend towards the use of ceramic heads. Thereby, the trunnion material plays an important role. Less rigid titanium alloy stems (e.g., Accolade TMZF stem (Stryker Orthopedics, Mahwah, New Jersey, USA)) were introduced to decrease stress shielding and femoral bone loss around prosthesis [11]. However, a less rigid trunnion leads to more micromotions, corrosion and gross trunnion failures [10]. Other implant-related risk factors were laser etching of the neck, introducer/extraction holes, metallurgic flaws, small-diameter necks and sharp corners at the neck.

### Patient-related factors

Patient-related risk factors are correlated to more intense use of the implant. The included patients had a relatively young age of 65 years at the time of fracture and most had an active lifestyle, leading to more micromotions and more crevice corrosion at the head-neck interface [12]. Also, male gender, high body weight and greater BMI were patient-related risk factors for implant fractures.

### Surgeon-related factors

One study reported that damage caused by the Hohmann retractors during revision surgery might have caused damage to the trunnion, leading to fracture. No other specific surgeon-related risk factors were mentioned in the other included studies.

In primary THA, the femoral head should be assembled well-centered on a clean and dry taper [13, 14]. Contaminated trunnions decrease the torsional resistance and increase fretting [15]. Ceramtec (CeramTec, AG, Plochingen, Germany) advises use of a single moderate hammer blow, which was in contrast to recent studies advising use of higher assembly forces to decrease wear [13, 16]. Higher assembly forces could increase the stability of the head-neck junction, but might damage the ceramic head. Generally, a 4 kN blow is advised.

In revision surgery, coaxial removal of the head, cleaning of the trunnion and removal of all corrosive products are important. Corrosive products on the trunnion will lead to a suboptimal head fit, causing micromotions and crevice corrosion. Placing back ceramic heads with a taper sleeve on damaged trunnions will lower the risk of ceramic fracture. During revision surgery, the femoral neck should be protected from scratches by the surgical instruments.

Moreover, the choice of material is an important factor in preventing corrosion. The use of ceramic heads instead of cobalt-chromium heads leads to lower wear rates [17]. Mixing and matching of components from different brands is discouraged by the manufacturers, because it could lead to a trunnion-head mismatch and thereby increased fretting and wear. Remarkably, a Dutch database study in 2016 did not find any differences in medium-term revision rates in the mixed-component groups [18]. However, the National Joint Registry of England and Wales showed higher failure rates if a head and a femoral stem from different manufacturers were used [19]. Fallahnezhad et al have shown a decreased torsional strength of the head-neck junction in the case of angular mismatch between the head and the neck connection [20]. Also, Mueller et al concluded that using head and necks from different manufacturers could lead to less taper connection strength [21]. However, with Corail (DePuy Synthes, Warsaw, IN, US) and Bicontact (B. Braun Aesculap, Tuttlingen, Germany) stems, mixing and matching led to a stronger connection, which was possibly attributed to better taper angles and higher quality femoral heads from competing companies [21]. Bitter et al found that taper mismatches, which could be caused by mixing and matching, led to more wear than a perfect fit, especially a tip fit is increasing the amount of wear [13]. Generally, mixing and matching different components could lead to an unstable head-neck connection and therefore we advise using head and stems from the same manufacturer to ensure a stable situation and reduce fretting and wear at the head-neck junction.

### Limitations

First, the low quality of evidence of the included studies did not allow us to perform statistical analysis. Few studies offered detailed information on the implant characteristics and microscopic analysis, which was not conducted in all cases. Another limitation of this review is that the conclusions are limited due to the relatively small number of identified cases. Finally, the restriction of our systematic review to include English, German, French and Dutch language studies may have resulted in language-related bias.

## Conclusion

In conclusion, femoral prosthesis neck fractures are a potentially increasing complication of THA. The mean time to neck fracture after femoral stem placement is 7 years. The fracture is etiologically multifactorial. Our conclusions are limited due to the relatively small number of identified cases, heterogeneity of subjects and low quality of included studies. In summary, a ceramic head must be placed on a clean and dry trunnion with an assembly force of 4 kN. In revision surgery, it is of great importance to prevent notching of the trunnion, clean and remove corrosive products before placing back a titanium sleeved ceramic head. Attentions should be paid to the type of alloy and it is desirable to use products from the same manufacturer. Finally, avoid the use of cobalt-chromium heads, especially on corroded trunnions.

## Data Availability

Not applicable.

## Abbreviations

THA: Total hip arthroplasty
IN, USA: Indiana, United States of America
PRISMA: Preferred Reporting Items for Systematic Reviews and Meta-Analyses
SD: Standard deviation
BMI: Body mass index
TMZF: Titanium alloy (Ti-12Mo-6Zr-2Fe)
kN: Kilonewton
